# African Primary Care Research: Current situation, priorities and capacity building

**DOI:** 10.4102/phcfm.v6i1.758

**Published:** 2014-12-05

**Authors:** Robert Mash, Akye Essuman, Riaz Ratansi, Felicity Goodyear-Smith, Klaus Von Pressentin, Zelra Malan, Marianne Van Lancker, Jan De Maeseneer

**Affiliations:** 1 Division of Family Medicine and Primary Care, Stellenbosch University, South Africa; 2 Department of Community Medicine, University of Ghana, Ghana; 3 Department of Family Medicine, Aga Khan University, Tanzania; 4 Department of General Practice and Primary Health Care, University of Auckland, New Zealand; 5 Department of Family Medicine, University of Ghent, Belgium

## Introduction

**FIGURE 1:**
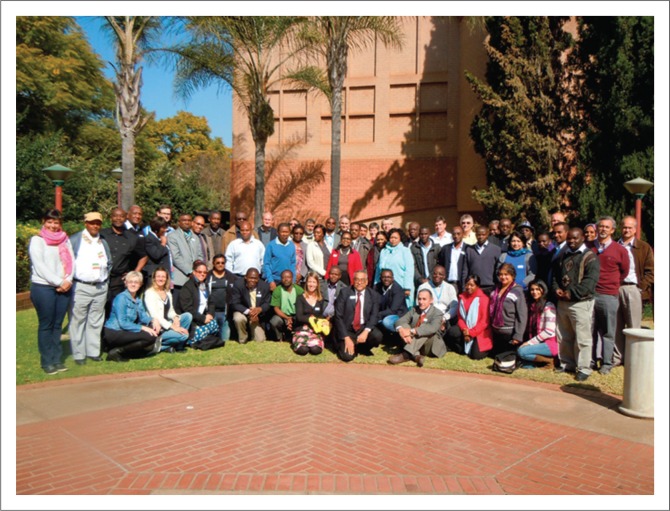
Delegates at the 6th PRIMAFAMED Workshop, 24 June 2014. *Source*: Photo taken by authors

The Sixth PRIMAFAMED (Primary Health Care/Family Medicine Education Network) workshop on ‘Capacity Building and Priorities in Primary Care Research’ was held in Pretoria, South Africa (SA), from 22 to 24 June 2014. Delegates from the following countries attended the workshop: Ghana, Nigeria, Uganda, Kenya, Tanzania, Sudan, Malawi, Zimbabwe, Botswana, Namibia, SA, Zambia, Ethiopia, Rwanda, Mozambique, Swaziland, Belgium, and Denmark (Figure 1). Delegates were from established or emerging departments of family medicine and primary care in these countries. The central theme of the workshop was primary care research – the current situation, the priorities for research and the need for capacity building. This report gives a summary of the consensus on these matters that emerged from the workshop.

The motivation for the conference was derived in part from the involvement of Professor Bob Mash (SA) and Professor Olayinka Ayankogbe (Nigeria) in the World Organization of Family Doctors (WONCA) Global Working Party on Primary Care Research, which has a goal of promoting primary care research.

### Process

**FIGURE 2:**
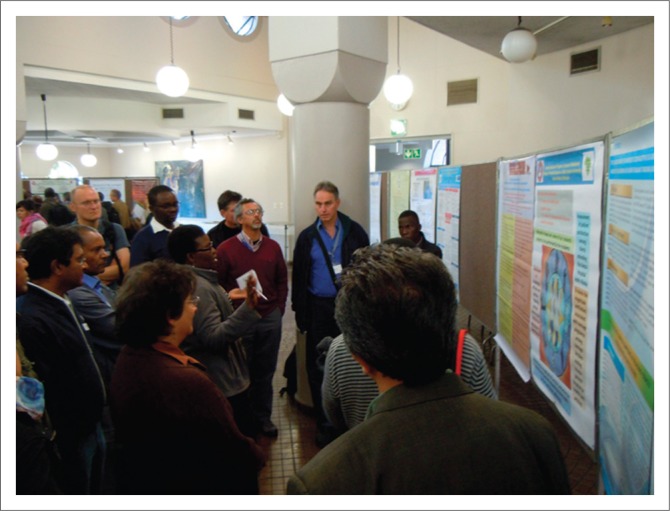
Delegates discuss the poster presentations. *Source*: Photo taken by authors

**FIGURE 3:**
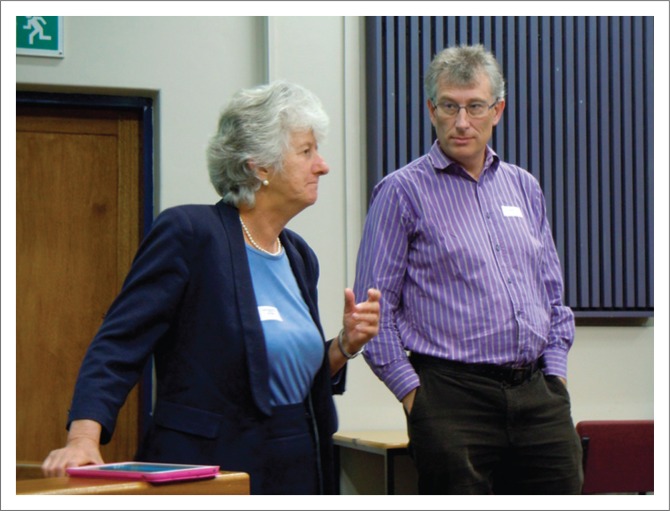
Professor Felicity Goodyear-Smith answers questions after her plenary address. *Source*: Photo taken by authors

**FIGURE 4:**
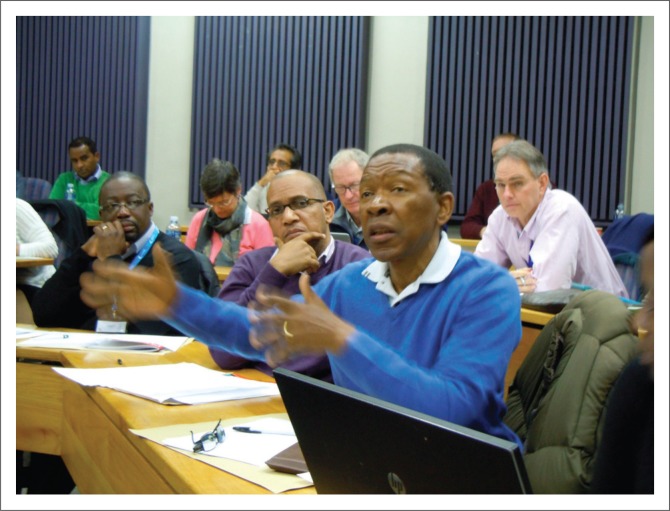
Final plenary discussion. *Source*: Photo taken by authors

A four-step process was followed leading up to this report on the final consensus:

1. Situational analysis: Each institution attending the workshop was requested to present a poster summarising their current research activities and output. The delegates reviewed these posters in an interactive poster session (Figure 2).
2. International perspective: Professor Felicity Goodyear-Smith addressed the conference on capacity building for primary care research (Figure 3) from her perspective as Head of Department of General Practice and Primary Health Care, University of Auckland; Founding Editor, Journal of Primary Health Care; Executive member, WONCA Working Party on Research; and Vice-Chair, International Committee, North American Primary Care Research Group.
3. Small group discussion: The delegates were divided into four groups to reflect on the situational analysis, give feedback on the current research priorities, define what capacity building was needed and give suggestions on how this capacity could be attained. Small groups were facilitated by Dr Akye Essuman (Ghana), Dr Riaz Ratansi (Tanzania), Prof Felicity Goodyear-Smith (New Zealand) and Prof Bob Mash (SA).
4. Consensus building plenary: Each of the four groups made a short Microsoft^®^ Powerpoint presentation in plenary and these presentations were followed by a general discussion (Figure 4). The comments and additional reflections made during the final plenary were documented.

This report is a summary of the final consensus achieved through this process.

### Situational analysis

The workshop considered the current strengths and weaknesses of primary care research in the African context from their perspective of the discipline of family medicine and primary care.

### Strengths of current situation

#### The context of family medicine and primary care researchers

Family medicine and primary care is a generalist discipline which works in communities, primary care facilities and district hospitals. Little research currently takes place within this context and there is therefore a huge potential for almost any research to be useful and to make a difference. Understanding community health needs and strengthening primary healthcare are important aspects of any country's health system. Research performed in communities and primary care can be more relevant with regard to people's health and the translation of evidence into practice. The research agenda is more closely aligned with the needs of communities. For example, the community can be seen as a ‘living laboratory’ and community-oriented primary care can result in rich data derived from both homes and families. Because of its generalist nature, primary care touches on issues across the full burden of disease and tends to be more person-oriented – trying to make sense of how people see health and disease. There is a clear opportunity for a partnership between service, training and research within a culture of learning in communities and primary care. The African context will also provide unique opportunities for primary care research that are not found elsewhere. As exemplified by the participants of this workshop, there is both interest in and commitment to increase capacity and activity in the area of primary care research.

#### Support for research activities is increasing on a small scale

Many of the institutions represented are increasingly offering support for research activities and capacity building – for example through the Medical Education Partnership Initiative (MEPI). Some institutions are putting pressure on their staff to perform better in the area of research. Universities, of course, also receive substantial funding and, in some countries, subsidies for research activities and outputs.

#### The region does have some leadership and expertise to support research

It should be acknowledged that the region does indeed contain some of the research expertise required to both support and enable primary care research.

#### Training programmes require students to perform research

Currently, postgraduate training in family medicine at most institutions requires students to perform research as part of their training. In a few cases, the undergraduate programme also prepares people for research activities.

#### Opportunities exist for publication and presentation of research

Within the region there are a number of national, regional and international journals, such as the *SA Family Practice Journal*, the *East African Medical Journal* and the *African Journal of Primary Health Care and Family Medicine*. In addition, there are a number of opportunities to present at national and regional conferences such as the annual SA National Family Practitioners’ Conference or the WONCA Regional Conference. Botswana is about to host its Second Family Medicine Conference.

#### There is an established culture of networking and collaboration

A number of networks and collaborations already exist in terms of developing training programmes through, for example, PRIMAFAMED and MEPI. There are also examples of research collaboration, such as with the Human Resources for Primary Care in Africa (HURAPRIM) project. This meeting itself demonstrated a huge potential for collaboration, not competition, between stakeholders. In some settings there may be opportunities for collaboration between the private and public sector.

#### There is an opportunity for interdisciplinary research teams

As the clinical nature of family medicine and primary care is to work in teams of community health workers, nurses, mid-level health workers, doctors and allied health professionals, there is an established culture of cooperation. This has the potential to enable interdisciplinary research approaches.

### Weaknesses in the current situation

Some delegates preferred to re-frame weaknesses as challenges and opportunities for future development.

#### Low research capability

Departments of family medicine and primary care have few academic staff and those that do exist often lack expertise in performing and supervising research. Most staff are either newly qualified or relatively junior and many postgraduate research projects are designed poorly or lack social and scientific value. Most research performed is descriptive and small scale and there is a lack of capacity to perform more experimental and analytical types of research on a larger scale.

#### Low research capacity – people, funding and resources

Large-scale funding is mostly from overseas donors and funding agencies and is not targeted at strengthening primary healthcare outside of certain priority diseases such as HIV and tuberculosis (TB). On the other hand, the lack of capability amongst researchers makes it difficult for them to compete for and obtain large-scale international funding; researchers may also fail to be aware of or take advantage of the smaller-scale grants and funding opportunities available locally. At this time, researchers should focus on low-cost, high-impact projects.

Some countries reported that they still have limited or unreliable access to the internet and key software and that their institutions could not afford access to many journals.

The demands of clinical service and teaching reduce the available time and energy for a focus on research. In addition, the number of postgraduate students at a Masters level to help drive research is also small in many countries.

#### Failure to publish and disseminate research findings

Despite the opportunities listed above, much of the research performed is not submitted for publication or presented at conferences. Research, however, should be judged not so much by the impact factor of the journal as by its impact on policy and practice, which may depend on strategies other than just publication.

#### High inertia in the system

The process of obtaining ethical approval and permission to perform research is a long and bureaucratic process in many institutions. This may be compounded by a lack of support for the types of research performed most commonly in primary care, for example qualitative and action research-type projects. Review committees and boards do not usually have representatives from the family medicine and primary care context.

#### Lack of innovation in types of research

People working in primary care may not see the rich opportunities for research that are a part of their daily work because of their prior exposure to types of research performed in referral hospitals, laboratories and clinical trials – which become normative in terms of their understanding of what research should be like. The opportunities for evaluation of community health needs, surveys, quality improvement studies, programme evaluation, participatory action and qualitative research are lost.

#### Poor coordination of research activities

Researchers often work on small-scale projects in isolation and without alignment to a clear set of local priorities. Few departments have a clearly agreed-upon research agenda.

#### Lack of collaboration in research activities

Despite the existing collaboration on training, there is relatively little collaboration between institutions and countries on primary care research projects. There is also a lack of awareness of the expertise and support that could be obtained from researchers within the same institutions, but from different disciplines. For example, there is no database of established researchers in the field and potential mentors.

#### Lack of support from academic and government policymakers

The relatively low status of family medicine and primary care in most universities and the hospital-centric view of many health systems, means that there is a relative lack of understanding and support from key leaders and stakeholders for primary care research. There is often no national plan or strategy for primary care research. On the other hand, researchers may also lack insight into the national research priorities that have been identified.

## Future priorities in primary care research

The delegates recognised that it is not possible to set specific priorities for the whole of Africa and that each country and institution must set its own such priorities for the local context. Nevertheless, some general comments and pointers were made based on the typology of primary care research suggested by John Beasley and Barbara Starfield.

### General comments

Primary care research should shift the focus from hospitals to primary healthcare and communities. Research should have clear social value to communities and scientific value to decision makers. In addition, research should use a mix of different methods and range across the whole of the typology outlined below.

#### Basic research

This should focus on the adaptation (e.g. of the primary care assessment tool) or development (e.g. family physician impact assessment tool) of key tools for use in primary care research in the African context.

#### Clinical research

Most research is currently in this domain. Research should focus across the whole local burden of disease (e.g. HIV/AIDS, TB, non-communicable chronic diseases, injury and violence, maternal and child mortality, etc.) and look at cost-effective interventions in order to improve the quality of care or community-oriented primary care.

#### Health services

There is currently little research looking at the core dimensions of effective primary healthcare – access, continuity, coordination, comprehensiveness and efficiency. This, however, should be a priority area in terms of strengthening the primary healthcare system. Strengthening the health information system within the district is also a priority.

#### Health systems

Most research at this level has been on the contribution of family medicine, family physicians and primary care doctors to the health system. As this is still a contested issue in most African countries, this remains a priority – evidence for the contribution of family medicine and how family physicians should be utilised within the district health system. A broader theme is that of research on the human resources for primary healthcare in the African context.

#### Educational research

As family medicine and primary care training programmes are in a state of design and development in many countries, the need for supportive research to guide this process remains a priority. For example, curriculum development and faculty development are key topics.

## How to build capacity for primary care research

The development of both capability and capacity were seen as being a maturation process over time and not just an issue of training. Given the rich primary care context and the growing number of role players, the building of their capacity may unlock a new stream of research activity.

### Contribution of regional and international networks in family medicine and primary care

South-South collaboration, as well as North-South, should be enabled by the existing networks such as PRIMAFAMED, WONCA and MEPI. These networks should enable the sharing of expertise, resources and tools for research, as well as published research from within the network. They could also be a way of sharing information on funding opportunities and grants. These networks should also encourage the emergence of joint projects and provide training opportunities. Mentors and mentees should be connected and a database of expertise and mentorship created. Regional meetings are an opportunity for networking, benchmarking between countries, training and strategic planning. Websites or list servers operated by these networks can be a means of disseminating information and resources and should also become more interactive. Those better off in terms of resources should take the lead and involve others.

### Contribution of the individual countries and academic institutions

#### Develop national policy which includes a focus on primary care research

Enable funding mechanisms for emerging primary care researchers. The subsidy scheme in SA by the Department of Education to universities linked to research outputs is a useful incentive and funding mechanism.

#### Universities should look at building formal links for primary care research

Universities and faculties should look at how orientation to and preparation for research is built into the undergraduate programmes (e.g. research toolbox, extra credit for research). Developing skills in evidence-based practice can complement the development of research capability. They should also create opportunities for the presentation and even in-house publication of research, with incentives and prizes for participation, thus encourage emerging researchers. In addition, universities and faculties should ensure that the process for ethics approval and permission to perform research is an efficient process that supports primary care research.

Accepting the research assignment for the MMed in the format of a journal article and incentivising publication as an option for assessment during the degree (i.e., do not need external examination if accepted for publication through peer review by an accredited journal) can be a further means of encouraging throughput and publication.

### Contribution of the departments of family medicine and primary care

Each department should develop a clear research agenda and strategy for capacity building, which can give direction to staff and students in terms of their research questions and topics. This should also be communicated to the broader faculty. It is important to ensure that student projects are aligned with this agenda and bring multiple small-scale individual projects together to make a larger, more integrated whole.

Departments should engage with the communities served when setting the research agenda as this will ensure more social accountability. In addition, they should collaborate with local research expertise (e.g. public health) in order to deliver on the research agenda set above. The possibility of interdisciplinary research teams should be explored, which would also encourage critical thinking from different perspectives.

Partnership with health services and policy makers would ensure that research is relevant and that findings will be incorporated into decision making.

It is essential to develop a research culture – reward and celebrate research outputs and link more experienced researchers with emerging researchers. In this way, it will be possible to integrate service, learning and research – *research what you do*.

Departments and researchers should make use of resources such as the 10 articles just published on primary care research methods in the *African Journal of Primary Health Care and Family Medicine*. In addition, they should ensure that they have registered with the journal and get e-alerts of published articles.

As well as making full use of local opportunities for training and funding, it would be worth considering having a designated primary care research champion who can link with others in the region and meet at WONCA or PRIMAFAMED.

### Training issues

Training needs can be met at all levels, for example, distance learning courses from the broader international community, training during PRIMAFAMED or WONCA meetings in the region, by the University or Faculty, or even within the specific department:

- Create opportunities for advanced research training through doctoral degree programmes. Aim for each -department to have at least one person with a PhD who is able to supervise and capacitate others. Look for funds to support this initiative, capacity for doctoral supervision and opportunities for training (e.g. Stellenbosch University African Doctoral Academy).
- Provide courses or retreats on scientific writing skills for proposals, grants, reports and publications.
- Provide courses on relevant methodologies for primary care researchers.

## Conclusion

This conference provided an opportunity for key role players from academic departments of family medicine and primary care in Africa to interact on the topic of building capacity for primary care research. Delegates collaborated on a situational analysis, discussed the current priorities and considered ways of building more capacity in the African context.

